# Association Between Preoperative Radiological Findings and Outcomes After Total Knee Arthroplasty

**DOI:** 10.7759/cureus.75697

**Published:** 2024-12-14

**Authors:** Abdulaziz F Bokhari, Leena Alwafi, Asim A Alrimy, Mouath Asiri, Mohammed M Bukhari, Abdulaziz S Alrashid, Abdulaziz A Alsiraihi, Jalal A Zahhar, Hassan O Bogari

**Affiliations:** 1 Medicine, King Saud Bin Abdulaziz University for Health Sciences College of Medicine, Jeddah, SAU; 2 Musculoskeletal Imaging, Ministry of National Gaurd Hospital Affairs, Jeddah, SAU

**Keywords:** knee arthroplasty, knee surgery outcomes, msk radiology, musculoskeletal imaging, total knee replacement (tkr)

## Abstract

Introduction

Total knee arthroplasty (TKA) is a widely accepted surgical intervention for patients with advanced knee osteoarthritis, aimed at reducing pain and improving functional mobility. Preoperative radiological evaluations, including assessments of joint space narrowing, osteophytes, varus/valgus deformities, and subchondral sclerosis, are essential for planning the surgery and predicting postoperative outcomes. Although extensive research has been conducted internationally, data focusing on populations in Saudi Arabia remain limited. This study investigates the association between preoperative radiological findings and postoperative outcomes, including pain, range of motion (ROM), and functional status, in a Saudi retrospective cross-sectional study.

Methods

This retrospective cross-sectional study was conducted at King Abdulaziz Medical City in Jeddah, Saudi Arabia. A total of 523 patients who underwent TKA were included. Data were collected from medical records through the BESTCare system, focusing on patient demographics, preoperative and postoperative radiological findings, and surgical outcomes. Statistical analyses were performed using IBM SPSS Statistics software (IBM Corp., Armonk, NY), and the significance was determined at p < 0.05.

Results

The study population had a median age of 64 years, and 72% were female. Preoperative radiological findings included varus deformity (65.39%), joint space narrowing (47.61%), and osteophytes (31.17%). Postoperative outcomes showed 19% of patients reported pain, 85% regained normal ROM, and 74% returned to normal functional status. Significant associations were found between postoperative limited ROM and preoperative osteophytes (p = 0.021), subchondral sclerosis (p = 0.033), and osteopenia (p = 0.017). Subchondral sclerosis was also linked to postoperative functional impairment (p = 0.009). No significant association was observed between preoperative radiological findings and postoperative pain or thromboembolism.

Conclusion

Preoperative radiological markers, particularly osteophytes and subchondral sclerosis, were significant predictors of postoperative ROM and functional status in TKA patients. Identifying these markers can enhance preoperative planning, enable targeted rehabilitation strategies, and improve patient outcomes in the Saudi population. Further studies are warranted to confirm these findings and explore additional predictive factors.

## Introduction

Total knee arthroplasty (TKA) is the definitive surgical treatment for advanced knee osteoarthritis, aimed at alleviating pain and restoring function [1]. As the prevalence of osteoarthritis increases globally, particularly due to aging populations and rising obesity rates, optimizing TKA outcomes has become increasingly important. Preoperative radiological evaluations are critical in this process, as they provide valuable insights into the structural and functional integrity of the knee joint. These assessments help guide surgical planning and can predict postoperative outcomes, including pain, range of motion (ROM), and long-term function. 

Existing literature has explored various preoperative radiological factors, such as joint space narrowing, osteophytes, varus/valgus deformities, and subchondral sclerosis, that impact the success of TKA [2-7]. For instance, Hu et al. (2022) highlighted the importance of preoperative knee alignment in predicting both the functional outcomes and the longevity of the prosthetic implant following TKA [2]. Their findings underscore the relevance of detailed preoperative radiological evaluations for patient-specific surgical planning. 

However, despite the abundance of international research on the correlation between preoperative radiological findings and TKA outcomes, there is a relative lack of data focusing on local populations in Saudi Arabia, where the demographic profile, including higher obesity rates and lifestyle factors, may influence TKA outcomes differently. This research seeks to bridge this gap by examining the association between preoperative radiological findings and postoperative outcomes. 

The primary goal of our research is to determine whether specific preoperative radiological findings, such as joint space narrowing, osteophytes, subchondral sclerosis, and varus deformities, are predictive of postoperative outcomes, including pain, ROM, and functional status. Additionally, this study aims to identify whether preoperative radiological markers are linked to an increased risk of postoperative complications, such as limited mobility or limited range of motion after TKA. Understanding these relationships can help optimize preoperative assessments, tailor surgical approaches, and improve patient-specific postoperative outcomes in TKA patients in Saudi Arabia.

## Materials and methods

The study was a retrospective cross-sectional analytic design aimed at assessing the association between preoperative radiological findings and postoperative outcomes after TKA. The study was at King Abdulaziz Medical City in Jeddah, Saudi Arabia, part of the National Guard Health Affairs. Data for this research were gathered through chart reviews utilizing a comprehensive review of patient medical records through the BESTCare system (internal automated medical records) to collect relevant data on postoperative outcomes and associated radiological findings. 

The population included 523 patients who underwent total knee replacement. Inclusion criteria encompassed all TKA patients, while exclusion criteria excluded patients with incomplete data. Convenience sampling was used to gather data from available patient records. The data included patient demographics, such as age, gender, and BMI, as well as both preoperative and postoperative radiological findings. Information on surgical details, including the type of surgery and the reason for surgery, was also collected, along with postoperative outcomes such as postoperative function and postoperative. 

For data management and analysis, the information was entered into Microsoft Excel (Microsoft Corporation, Redmond, WA, USA) for cleaning and verification. Statistical analysis was performed using IBM SPSS Statistics software (IBM Corporation, Armonk, NY, USA). Descriptive statistics summarizing patient demographics and baseline characteristics, with categorical variables being presented as frequencies and percentages and continuous variables as means with standard deviations. Chi-square or Fisher’s exact tests were employed to compare categorical variables, while t-tests or ANOVA were used for continuous variables. A p-value of less than 0.05 was considered statistically significant. 

Ethical approval was obtained from the relevant institutional review board (IRB), and patient confidentiality was maintained throughout the study. The study was approved by the King Abdullah International Medical Research Center (KAIMRC) with study approval number NRJ24/051/8.

## Results

The study included 523 patients who underwent total knee replacement. The median age was 64 years, with 72% (n=376) being females. Only 4.4% (n=23) had a normal weight, 19% (n=98) were overweight, and 77% (n=402) were obese (Table 1).

**Table 1:** Demographic characteristics of patients with total knee replacement ^1^n (%); Median (IQR); *multi-answer question; N = 523^1^

Characteristic	N = 523^1^
Gender	
Female	376 (72%)
Male	147 (28%)
Age (years)	64 (60, 71)
Body mass index (kg/m^2^)	
Normal weight	23 (4.4%)
Overweight	98 (19%)
Obese	402 (77%)

Regarding the type of surgery, 71% (n=370) of patients underwent unilateral procedures, while 29% (n=153) had bilateral surgeries. Preoperative functional quality of life varied, with 57% (n=259) of patients considered to have normal function, 32% (n=146) of patients requiring assistance, and 11% (n=48) of patients using a wheelchair. Preoperative ROM was limited in 58% (n=255) of patients and normal in 42% (n=181) (Table 2).

**Table 2:** Preoperative characteristics of patients with total knee replacement ^1^n (%); Median (interquartile range (IQR)); N = 523^1^

Characteristic	N = 523^1 ^
Surgery type	
Unilateral	370 (71%)
Bilateral	153 (29%)
Preoperative functional quality of life	
Normal	259 (57%)
Needs assistance	146 (32%)
On wheelchair	48 (11%)
Preoperative range of motion (ROM)	
Limited	255 (58%)
Normal	181 (42%)

**Figure 1:**
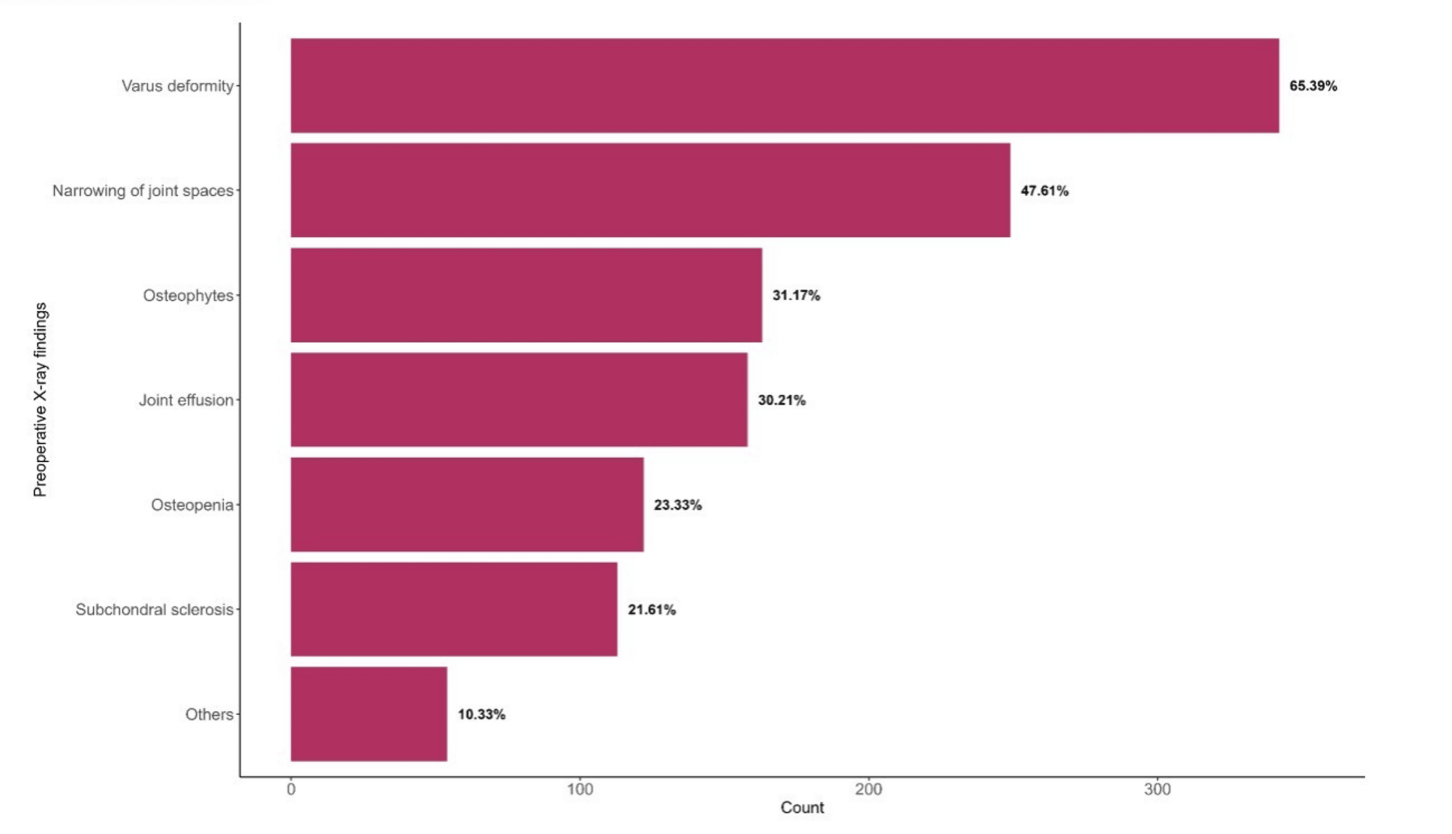
Preoperative X-ray finding of patients with total knee replacement N=523^1^

The most common preoperative X-ray finding among patients was varus deformity observed in 65.39% (n=342) of cases, followed by a narrowing of joint spaces (47.61%, n=249), osteophytes (31.17%, n=163), joint effusion (30.21%, n=158), osteopenia (23.33%, n=122), subchondral sclerosis (21.61%, n=113), and others (10.33%, n=54) (Figure 1).

Postoperative pain was reported by 19% (n=101) of patients, while postoperative ROM was normal in 85% (n=445) and limited in 15% (n=78). In terms of postoperative function, 74% (n=386) of patients regained normal function, 22% (n=115) required assistance, and 4.2% (n=22) were wheelchair-bound. Thromboembolism occurred in 1.7% (n=9) (Table 3).

**Table 3:** Postoperative characteristics of patients with total knee replacement ^1^n (%): Median (interquartile range (IQR)); N = 523^1^

Characteristic	N = 523^1 ^
Postoperative pain	
No	422 (81%)
Yes	101 (19%)
Postoperative range of motion (ROM)	
Limited	78 (15%)
Normal	445 (85%)
Postoperative function	
Normal	386 (74%)
Needs assistance	115 (22%)
On wheelchair	22 (4.2%)
Thromboembolism	
No	521 (98.2%)
Yes	9 (1.7%)

Postoperative pain and postoperative thromboembolism were not associated with preoperative radiological findings (p > 0.05) (Tables 4-5). Significant preoperative radiological findings that were associated with postoperative limited range of motion were osteopenia (p = 0.017), osteophytes (p = 0.021), and patients with subchondral sclerosis (p = 0.033) (Table 6). Moreover, the preoperative radiological finding of subchondral sclerosis was significantly associated with the postoperative need for assistance and a wheelchair (p = 0.009) (Table 7). 

**Table 4:** Association between postoperative pain and preoperative radiological findings ^1^n (%); ^2^Pearson's chi-squared test; p<0.05

	Postoperative pain			
Characteristic	No, N = 422^1^	Yes, N = 101^1^	Chi-square value	p-value^2^
Joint effusion			0.00	>0.9
No	294 (70%)	71 (70%)		
Yes	128 (30%)	30 (30%)		
Narrowing of joint spaces			1.5	0.2
No	215 (51%)	59 (58%)		
Yes	207 (49%)	42 (42%)		
Osteopenia			0.08	0.7
No	322 (76%)	79 (78%)		
Yes	100 (24%)	22 (22%)		
Varus deformity			0.13	0.6
No	144 (34%)	37 (37%)		
Yes	278 (66%)	64 (63%)		
Others			0.57	0.3
No	381 (90%)	88 (87%)		
Yes	41 (9.7%)	13 (13%)		
Osteophytes			0.00	>0.9
No	291 (69%)	69 (68%)		
Yes	131 (31%)	32 (32%)		
Subchondral sclerosis			0.52	0.4
No	334 (79%)	76 (75%)		
Yes	88 (21%)	25 (25%)		

**Table 5:** Association between postoperative thromboembolism and preoperative radiological findings ^1^n (%); ^2^Fisher's exact test; p<0.05

	Thromboembolism		
Characteristic	No, N = 514^1^	Yes, N = 9^1^	p-value^2^
Joint effusion			0.7
No	358 (70%)	7 (78%)	
Yes	156 (30%)	2 (22%)	
Narrowing of joint spaces			0.3
No	271 (53%)	3 (33%)	
Yes	243 (47%)	6 (67%)	
Osteopenia			0.2
No	396 (77%)	5 (56%)	
Yes	118 (23%)	4 (44%)	
Varus deformity			0.5
No	177 (34%)	4 (44%)	
Yes	337 (66%)	5 (56%)	
Others			0.2
No	462 (90%)	7 (78%)	
Yes	52 (10%)	2 (22%)	
Osteophytes			0.5
No	355 (69%)	5 (56%)	
Yes	159 (31%)	4 (44%)	
Subchondral sclerosis			0.11
No	405 (79%)	5 (56%)	
Yes	109 (21%)	4 (44%)	

**Table 6:** Association between postoperative range of motion and preoperative radiological findings ^1^n (%); ^2^Pearson's chi-squared test; p<0.05

	Postoperative range of motion			
Characteristic	Limited, N = 78^1^	Normal, N = 445^1^	Chi-square value	p-value^2^
Joint effusion			0.47	0.5
No	57 (73%)	308 (69%)		
Yes	21 (27%)	137 (31%)		
Narrowing of joint spaces			0.08	0.8
No	42 (54%)	232 (52%)		
Yes	36 (46%)	213 (48%)		
Osteopenia			5.7	0.017
No	68 (87%)	333 (75%)		
Yes	10 (13%)	112 (25%)		
Varus deformity			2.4	0.12
No	33 (42%)	148 (33%)		
Yes	45 (58%)	297 (67%)		
Others			2.5	0.11
No	66 (85%)	403 (91%)		
Yes	12 (15%)	42 (9.4%)		
Osteophytes			5.3	0.021
No	45 (58%)	315 (71%)		
Yes	33 (42%)	130 (29%)		
Subchondral sclerosis			4.5	0.033
No	54 (69%)	356 (80%)		
Yes	24 (31%)	89 (20%)		

**Table 7:** Association between postoperative function and preoperative radiological findings ^1^n (%); ^2^Pearson's chi-squared test; Fisher's exact test; p<0.05

	Postoperative function				
Characteristic	Need assistance, N = 115^1^	Normal, N = 386^1^	On wheelchair, N = 22^1^	Chi-square value	p-value^2^
Joint effusion				0.09	>0.9
No	80 (70%)	269 (70%)	16 (73%)		
Yes	35 (30%)	117 (30%)	6 (27%)		
Narrowing of joint spaces				1.6	0.5
No	58 (50%)	207 (54%)	9 (41%)		
Yes	57 (50%)	179 (46%)	13 (59%)		
Osteopenia				0.52	0.8
No	91 (79%)	293 (76%)	17 (77%)		
Yes	24 (21%)	93 (24%)	5 (23%)		
Varus deformity				2.0	0.4
No	46 (40%)	127 (33%)	8 (36%)		
Yes	69 (60%)	259 (67%)	14 (64%)		
Others				5.1	0.064
No	97 (84%)	353 (91%)	19 (86%)		
Yes	18 (16%)	33 (8.5%)	3 (14%)		
Osteophytes				2.2	0.3
No	80 (70%)	268 (69%)	12 (55%)		
Yes	35 (30%)	118 (31%)	10 (45%)		
Subchondral sclerosis				10.9	0.009
No	92 (80%)	307 (80%)	11 (50%)		
Yes	23 (20%)	79 (20%)	11 (50%)		

## Discussion

In this study, we aimed to investigate the association between preoperative radiological findings and postoperative outcomes in patients undergoing TKA at King Abdulaziz Medical City in Saudi Arabia. Our primary goal was to investigate whether specific radiological findings can predict postoperative pain, ROM, functional status, and thromboembolism. By understanding these associations, we can help improve preoperative assessments, predict postoperative outcomes, and personalize postoperative management plans. 

Our cohort included 523 patients; the median age was 64 years old, consisting mainly of females (72%), and the majority of patients were obese (77%). It is worth noting that these demographics are important to consider since it is well-established that obesity is a risk factor for the development of osteoarthritis, which could also affect the outcomes of TKA and the generalizability of our study. Preoperatively, varus deformity was the most common radiological finding (65.39%), followed by joint space narrowing (47.61%), osteophytes (31.17%), joint effusion (30.21%), osteopenia (23.33%), and subchondral sclerosis (21.61%). These findings are characteristic of advanced osteoarthritis, as described in previous studies [8-9]. Notably, 58% of patients had limited ROM before surgery, and only 57% had normal functional quality of life, highlighting substantial impairment. 

Postoperatively, 19% of patients reported pain, 85% achieved normal ROM, and 74% regained normal function. Thromboembolism occurred in 1.7% of patients. Importantly, certain preoperative radiological findings showed significant associations with postoperative outcomes. 

Association with the postoperative ROM

The presence of osteophytes and subchondral sclerosis was significantly associated with limited postoperative ROM (p = 0.021 and p = 0.033, respectively). Osteophytes indicate the formation of bone spurs, which are common in advanced osteoarthritis and can limit joint mobility. Subchondral sclerosis reflects increased bone density beneath the cartilage surface, often resulting from chronic stress and degeneration [9]. These findings suggest that patients with these radiological markers may have more severe joint degeneration, leading to poorer postoperative ROM. 

Osteopenia was associated with a better ROM in the postoperative period (p=0.017). This unexpected result may be due to biases or confounding factors rather than a direct causal relationship. For instance, patients with osteopenia might differ in certain characteristics such as age, gender, or overall health status, which can influence postoperative recovery, management, and ROM. The group of patients who had osteopenia may have been receiving slightly different management or may have been more compliant with rehabilitation protocols. Another possibility is that the time at which ROM is measured postoperatively could lead to inaccurate results [10]. These biases could skew the result in such a way that a condition such as osteopenia is depicted to be beneficial when it may not be. Further research controlling for these potential biases is necessary to clarify this relationship. 

Association with postoperative function 

Subchondral sclerosis was also significantly associated with postoperative functional status, specifically the need for assistance or wheelchair use (p=0.009). Patients with subchondral sclerosis were more likely to have impaired function postoperatively. All other radiological features investigated were not significantly associated with postoperative function. 

Lack of association with postoperative pain and thromboembolism 

None of the preoperative radiological findings investigated showed a significant association with postoperative pain or thromboembolism. Pain perception is multifactorial, influenced by factors such as patient expectations, pain tolerance, and psychological state [11]. Thromboembolism was noted to have occurred in 1.7% of patients, which is within the expected range for TKA procedures [12]. Thromboembolism risk is more closely related to perioperative management and patient comorbidities rather than radiological findings [13]. 

Clinical implications 

The associations identified have practical implications.

Enhanced Preoperative Assessment

Enhanced assessment prior to surgery allows treating physicians to identify patients with osteophytes and subchondral sclerosis and can help anticipate potential challenges in postoperative recovery. 

Targeted Rehabilitation

Recognizing patients at risk for limited ROM and functional impairment in advance allows for the implementation of specialized physiotherapy programs during the postoperative period. 

Patient Education

Informing patients of their specific risks could lead to better expectations and potentially improve patients’ satisfaction with outcomes. 

Specialized Surgical Interventions

Patients identified with enhanced preoperative assessment could prompt surgeons to slightly adjust the management plan by using specific implants or considering aggressive debridement. 

Comparison with existing literature 

The literature is somewhat lacking in studies investigating preoperative radiological findings and their association with TKA postoperative outcomes. A study by Lange et al. [14] found no association between postoperative outcomes and preoperative radiological osteoarthritis severity. 

However, our study contrasts with Lange et al.’s findings by demonstrating specific associations between certain radiological markers, such as osteophytes and subchondral sclerosis, and poorer postoperative outcomes, particularly in ROM and functional status. The study by Toguchi et al. [3] showed postoperative outcomes were generally better in patients with severe varus deformities when osteophytes were minimal, while no significant differences were noted in patients with mild varus deformities regardless of osteophyte size. In contrast, our study showed no significant difference between varus deformities and postoperative outcomes, which could be attributed to differences in how varus deformity was measured. Toguchi et al. specifically analyzed severe and mild varus deformities separately, whereas our data did not make this distinction, possibly explaining the discrepancy in results. Future research should aim to separate and analyze mild and severe varus deformities to capture these nuanced differences. These discrepancies in our results and the studies mentioned could also be due to differences in study populations or the timing of outcome assessments. Moreover, our finding on osteopenia's unexpected association with improved postoperative ROM highlights the complexity of predicting surgical outcomes based solely on preoperative imaging and the need for standardized preoperative radiological criteria that can reliably predict postoperative outcomes. Future research is needed to validate our findings in larger, more diverse populations and should aim to control for confounding factors and variations in surgical techniques and consider analyzing mild and severe varus deformities separately.

Limitations 

Several limitations of our study must be considered. Firstly, the retrospective design of our study may introduce bias and limit our ability to draw causal inferences from the data. Furthermore, missing data in our cohort could affect the reliability of the results. Uncontrolled variables such as differences in surgical techniques, different prosthesis types, and rehabilitation programs may have influenced the outcomes. Lastly, the study's population homogeneity may limit the generalizability of the findings to other populations.

## Conclusions

In conclusion, this study demonstrated that preoperative radiological findings play a significant role in predicting outcomes after TKA. Factors like the presence of osteopenia, osteophytes, and patients with subchondral sclerosis showed significant correlation with postoperative limited range of motion. Moreover, the preoperative radiological finding of subchondral sclerosis was significantly associated with the postoperative need for assistance and a wheelchair. Interestingly, while we anticipated osteopenia to be mainly associated with postoperative limited range of motion, it did show a statistically significant association with normal ROM and limited ROM in this cohort. These findings suggest focusing on the specific radiological indicators that are most predictive of outcomes. Future research should aim to further investigate these associations to refine surgical planning and optimize patient care in TKA. 
